# Vaginal Calculus Formation on Exposed Midurethral Sling Mesh

**DOI:** 10.1155/2024/8287400

**Published:** 2024-03-16

**Authors:** Adam J. Langer, Zenab Saeed, Elizabeth Barrett, Rose A. Maxwell, David N. Dhanraj, Geoffrey D. Towers, Eyra A. Agudu

**Affiliations:** ^1^Department of Obstetrics and Gynecology, Wright State University, Dayton, USA; ^2^Boonshoft School of Medicine, Wright State University, Dayton, USA

## Abstract

The presence of a vaginal calculus is a rare clinical entity which may develop in the setting of vaginal urinary stagnation. Numerous factors contribute to stone formation, and management can be complicated by variations in size, location of the stone, and location of adjacent structures. Generally, once diagnosed, vaginal calculi should be removed and surrounding anatomy should be evaluated thoroughly for secondary fistula, erosion, or presence of an instituting foreign body. This report presents a case of vaginal calculus formation on exposed midurethral sling mesh in an elderly patient with hemorrhagic cystitis. This report emphasizes contributing pathophysiology, diagnostic factors, and treatment.

## 1. Introduction

Vaginal calculi are a rare gynecological finding [1, 2]. Stones, which vary in size, usually present as singular and may have a range of presenting symptoms [1–6]. Calculi are classified as either primary or secondary. Primary stones result from intravaginal urinary stasis in the context of aberrant anatomic features, including congenital genitourinary anomalies, trauma, fistulae, or obstructions [1, 2]. Risk factors for development of primary stones can include conditions associated with vaginal urinary stasis, such as neuropathic bladder, bladder exstrophy, myelodysplasia, cerebral palsy, mental delays, and vaginal outlet obstructions [7]. Based on the etiology of underlying stone formation, primary calculi more commonly occur in younger patients with congenital anomalies or elderly patients with fistulas or strictures [1].

Secondary stones occur due to deposition of urine salts related to foreign bodies. This represents the second most common cause of stone formation, following primary stones due to fistulae in the setting of urinary tract infections [3]. In secondary calculi, a foreign body can serve as the stone's nucleus around which urinary salt deposition precipitates [1].

Regardless of stone classification, the underlying cause of stone formation is urine stagnation and urease-positive bacterium involvement [1, 2]. Genitourinary microbes like E. coli, Proteus mirabilis, and Klebsiella species have been shown to play a role in stone formation [7], leading to alkalization of the physiologically acidic vaginal environment [2].

The present case involves a secondary vaginal calculus, adherent to the anterior vaginal wall. This was likely a result of midurethral sling mesh exposure in the setting of chronic urinary incontinence and concurrent infectious hemorrhagic cystitis in an elderly patient.

## 2. Case Illustration

The patient is a 74-year-old G4P4 female with history of total vaginal hysterectomy and midurethral sling performed 5-7 years prior to presentation for postmenopausal bleeding and stress urinary incontinence. She presented with new-onset postmenopausal vaginal bleeding described as passage of numerous small clots, along with sudden onset of vaginal pressure. Her history was notable for four prior vaginal births, morbid obesity with BMI of 71.97 kg/m^2^, atrial fibrillation on apixaban, hyperlipidemia, history of breast cancer with unilateral mastectomy, hypothyroidism, pulmonary hypertension, chronic diastolic congestive heart failure, depression, stage 3b kidney failure, and lymphedema.

**Figure 1:**
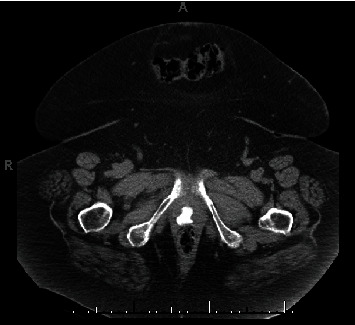
Axial CT scan image of the pelvis showing 2.16 × 1.76 cm calcified vaginal mass.

**Figure 2:**
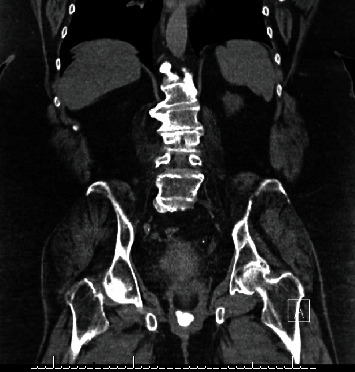
Coronal CT scan image of pelvis showing calcified vaginal mass.

The initial pelvic exam, though limited due to body habitus and mobility, showed a grey-white, rough-appearing mass at the vaginal introitus that was firm on bimanual exam in addition to malodorous bloody discharge. Initial laboratory findings showed leukocytosis without anemia. The preliminary report from an outside hospital abdominal CT scan showed concern for a 14 × 11 cm mass in the pelvis and mild hydronephrosis bilaterally. This was later determined to be distended bladder secondary to hemorrhagic cystitis. CT scan also showed a calcified vaginal versus bladder mass (Figures 1 and 2).

**Figure 3:**
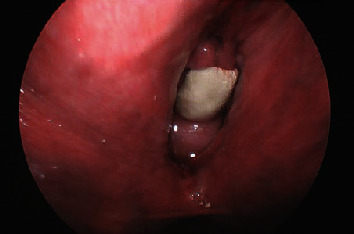
Vaginal stone adherent to anterior vaginal wall, at the level of the midurethra.

**Figure 4:**
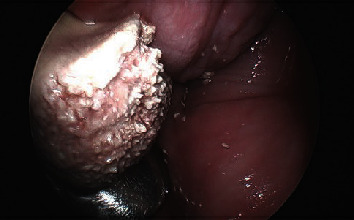
Magnified image of vaginal stone.

**Figure 5:**
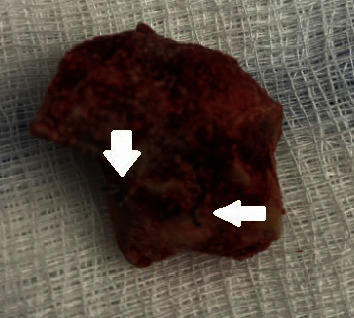
Excised vaginal stone with midurethral sling mesh fibers, on the side that was adherent to the anterior vaginal wall.

The patient underwent a pelvic exam under general anesthesia with cystourethroscopy and resection of the vaginal mass. Gynecologic oncology and urology were consulted intraoperatively due to concern for malignancy and urethral/bladder involvement, respectively. The calcified vaginal mass was identified and found to be adherent to the anterior vaginal wall; this was excised sharply at the level of the midurethra (Figures 3 and 4). Underlying the calcified mass was exposed mesh from her prior midurethral sling procedure (Figure 5). A small portion of the underlying mesh was excised, and the surrounding mucosa was reapproximated with delayed absorbable sutures. On cystourethroscopy, there was no evidence of fistula or injury to the urethra or bladder. Cystoscopic findings were consistent with hemorrhagic cystitis, showing gross bladder mucosal inflammation and an intact urethra without diverticulum or erosion. A large amount of clot and blood was evacuated from the bladder. Surgical pathology of the vaginal mass revealed a white/tan, firm, calcified mass with visible mesh measuring 2.4 × 2.0 × 1.7 cm consisting of amorphous material and calcifications without viable epithelium.

The patient developed an intraoperative fever, and broad-spectrum antibiotic therapy was initiated and continued for 48 hours. She was then transitioned to IV ceftriaxone which was continued until discharge. She did not have any additional fevers for the remainder of her hospitalization. Blood and urine cultures were positive for *Proteus mirabilis*. The patient's Foley catheter was removed on hospital day 6, and she was discharged to an extended care facility and transitioned to oral cefuroxime. She was then lost to follow-up. She did see her cardiologist one month after this admission and denied any recurrence of vaginal bleeding.

## 3. Discussion

Although vaginal calculi in the context of mesh exposure are uncommon, numerous case reports have demonstrated stone formation in various clinical contexts. The earliest report of a case involving a vaginal calculus, documented in 1900, showed stone formation in a vaginal cystocele [6].

Primary stones, which are most common and often due to fistulae, have been documented in numerous cases including a pediatric patient with a partial vaginal outlet obstruction resulting in poor urinary drainage from the vagina leading to calculus formation [8] and a patient with a urogenital sinus anomaly [5].

Secondary stone formation, typically preceded by urine stagnation in the vagina [5, 9], is exacerbated due to precipitation on a foreign body. Prior case reports of secondary vaginal calculi, as in the current case, have shown stone formation on vaginal tape mesh [10], an intrauterine contraception device [11], and a pessary [12]. Few previous cases have reported stone formation on mesh [3, 9], but exposure of mesh to urine serves as a nidus for stone formation. Synthetic mesh has been used surgically since 1950 and adapted for use in gynecological surgeries since 1970 [13]. Mesh is commonly used in urogynecology and has potential to serve as a nidus for secondary vaginal calculus formation [14]. Significant increases in reported adverse events related to vaginal mesh used in gynecological surgeries, including pelvic pain, infection, bleeding, urinary problems, and erosion or perforations, have led the Food and Drug Administration to strengthen requirements for mesh used in surgical cases [13]. For patients with gynecological foreign bodies, follow-up care and regular exams may be helpful in early detection of stones [9, 11]. Guidelines from the National Institute of Health and Care Excellence recommend follow-up for patients undergoing gynecological surgeries involving mesh to assess for complications, including indications for vaginal exam to assess mesh patency, exposure, or extrusion [15]. As in the presented case, however, mesh-associated complications like calculus formation may not present clinically until many years following initial surgery.

Stone formation has also been documented in patients with disability limiting ambulation, including multiple cases of paraplegia [7, 16]. In mobility-limiting conditions, chronic recumbency may increase urinary pooling in the vagina, leading to stasis and predilection for stone formation. This was seen in our case as the patient's morbid obesity had significantly limited her mobility.

Due to significant variation in presenting symptoms, diagnosis is often delayed or missed [2]. Such symptoms may include dyspareunia [5, 6], amenorrhea [5], abdominal pain [5], infertility [4], urinary urgency [2], urinary frequency [2], bleeding [2, 3], odor [2], pain [3], dysuria [3], and discharge [3]. Vaginal bleeding, odor, and pain brought the patient described above into the Emergency Department for initial evaluation. Depending on a stone's location in relation to the rectum, presenting symptoms may also include anal pain, stool deformation due to stone impingement on the rectum, and defecatory urgency [2]. Asymptomatic presentations or incidentally found stones have also been noted [3, 7, 11], and stone detection may be delayed until stone size causes symptom development [2] or even is palpable by the patient [3].

Diagnosis requires a thorough history and physical evaluation [2]. In terms of diagnostic imaging, vaginal calculi of the urate variety may require ultrasound imaging or direct visualization rather than X-ray radiography due to radiolucent properties [6]. Most stones, however, are struvite stones formed by urease-producing bacteria [3] and are seen as radiopaque on X-ray and CT.

Treatment generally includes surgical removal. In one case, stone removal was performed in an office setting [3]. Larger stones may necessitate an operating room or alternative extraction methods such as lithotripsy [16]. Surgical intervention should be mindful of stone adhesion to the vaginal wall or other genitourinary structures [2, 6]. Postremoval, patients should be evaluated for fistulas and mesh erosions [3]. In cases of primary stones due to anatomic variation, treatment may also necessitate surgical reconstruction of the vagina or urethra [5]. Certain microbes including E. coli, Proteus mirabilis, and Klebsiella species are associated with stone formation [7]. In the present case, the patient's urine cultures were positive for Proteus species, consistent with this proposed pathogenic role.

Although she was lost to follow-up upon discharge, the patient was recommended to schedule routine visits with her gynecologist, urologist, and primary care physician to prevent recurrent stone formation. Her gynecologist should perform regular pelvic exams to assess for further erosion of mesh that remains in place and should consider alternative methods of managing her urinary incontinence. Her urologist should repeat urine cultures to ensure adequate treatment of infection and could consider prophylactic antibiotics to prevent recurrent infection. The patient's primary care provider should seek to optimize her weight and other comorbidities contributing to her lack of mobility.

## 4. Conclusion

This case report illustrates a large vaginal calculus formation on exposed midurethral sling mesh in an elderly immobile patient. The cause of this uncommon complication in her case is multifactorial and includes midurethral sling mesh erosion, chronic urinary incontinence, chronic urinary stasis due to immobility and body habitus, lack of follow-up with gynecologist, and acutely hemorrhagic cystitis secondary to Proteus mirabilis urinary tract infection.
